# Why is a new journal dedicated to vascular biology required?

**DOI:** 10.1530/VB-18-0005

**Published:** 2019-01-03

**Authors:** Paolo Madeddu

**Affiliations:** 1 University of Bristol, Bristol, UK

The year 2018 marked the 110th anniversary of Goldmann’s discovery that vascularization is an active process in tissues (1) and the 50th anniversary of the concomitant reports from Greenblatt and Shubik (2) and Ehrmann and Knoth (3) that soluble morphogenic factors are required for cancer angiogenesis. Many other radically transformative paradigms have been introduced in the last decades. To name a few, the molecular search for the identity of master regulators of vascular tone led to the discovery of the endothelium-derived relaxing factor (EDRF; i.e. NO (4)), while clinically inspired investigations led to the recognition of the pathophysiological relevance of neoangiogenesis in cancer and tissue healing. This brought about the proposal of blocking angiogenesis to halt tumor growth and stimulating angiogenesis to treat myocardial ischemia and heart failure (5, 6, 7).

Importantly, recent years have seen a further dissection of vascular cell complexity and heterogeneity. In reality the concept that endothelial cells are heterogeneous is by no means new. In the 1950s and 1960s, electron microscopic studies showed structural differences between capillaries in different organs. In the 1980s, immunohistochemical studies revealed differential expression of lectins and other vascular antigens in the endothelium of various organs. More recently, the use of novel genomic and proteomic techniques has uncovered site- and single-cell-specific properties of the endothelium (8). The next challenge is to link the expressional diversity with functional diversity. To control each function, the vasculature exploits a remarkably flexible sensory system capable of intercepting simultaneous signaling from the circulation. McCarron *et al.* propose the fascinating theory that groups of neighboring endothelial cells can specialize in a distinctive sensory capacity and communicate the information on a large scale to other differently specialized cells. This organization, similar to swarm intelligence, provides a system-level sensing substantially greater than the capabilities of any single cell and more sophisticated than a scenario where all endothelial cells would be equally sensitive to extracellular cues (9).

Endothelial and mural cells display the striking capability to switch from a quiescent state to a highly active state. This switch has long been associated with changes in the levels of growth factors and other chemical signals. Recent findings show that changes in metabolism may even override signals inducing neoangiogenesis, and this may have important consequences under pathological conditions, such as diabetes and atherosclerosis. Moreover, vascular cells release chemokines and genetic material that affects the metabolism of other cell types in the same and distant tissue. Exciting new research focuses on vascular metabolism-centric therapeutic avenues for the cure of cardiovascular and tumoral diseases (10).

Vascular biology is a multidisciplinary field with research strands intersecting with many scientific and medical domains. This is increasingly becoming the case as the field continues to evolve and as more significant advances are made. A limiting aspect of basic research is that investigators often tend to tackle the different elements of the same problem as completely separate, dealing with each in a sequence, not worrying how their ‘solution’ is compatible with other interconnected problems. A more holistic and multidisciplinary approach may allow a quicker translation of basic vascular research findings into clinically valuable outcomes. *Vascular Biology* is a new journal which aims to provide a powerful platform bringing this research together, as opposed to publishing in many disparate overly niche or broad journals. The unrestricted access offered by open-access publication facilitates the widest possible dissemination of research findings, accelerating discovery, therapeutics and education across the many disciplines that vascular biology impacts.

*Vascular Biology,* brought to you by Bioscientifica, a long-established society publisher, is an open-access journal which will publish quality basic, clinical and translational research and reviews across all related disciplines, including cardiology, oncology, vascular development, inflammation, wound healing and bioengineering. Manuscripts will be published online shortly after acceptance and the final version of record will be published as soon as it is ready, which means papers will be citable immediately. We anticipate that *Vascular Biology* will be included in PubMed Central and Journal Citation Reports (Science Citation Index Expanded) as soon as the required number of articles have been published, at which point indexing will be applied to all articles retrospectively.

The journal is truly international, with an editorial board which includes representatives from around the globe. Expert strategic editors provide guidance and advice to the editorial team, and the world-class tier of senior editors have expertise across a broad range of subject areas, and are responsible for overseeing the peer review of submissions to the journal.

We strive to encourage collaboration for the benefit of biomedical science as a whole, and we are keenly aware of the need for an interdisciplinary approach to both clinical and basic research. Please submit your best work to the journal and show your support for this new endeavor. Publication in *Vascular Biology* will be free for all articles during the launch years, offering authors far-reaching exposure and impact for their work at no additional cost.

We look forward to receiving your submissions.

## Declaration of interest

The author declares that there is no conflict of interest that could be perceived as prejudicing the impartiality of this editorial.

## Funding

This work did not receive any specific grant from any funding agency in the public, commercial, or not-for-profit sector.
